# Structural and biochemical characterization of the cell fate determining nucleotidyltransferase fold protein MAB21L1

**DOI:** 10.1038/srep27498

**Published:** 2016-06-08

**Authors:** Carina C. de Oliveira Mann, Reiner Kiefersauer, Gregor Witte, Karl-Peter Hopfner

**Affiliations:** 1 Ludwig-Maximilians-Universität München, Gene Center and Dept. of Biochemistry, Feodor-Lynen-Str. 25, 81377 Munich, Germany; 2 Proteros Biostructures GmbH, Bunsenstraße 7a, 82152 Martinsried, Germany; 3 Center for Integrated Protein Science (CIPSM), Ludwig-Maximilians Universität München, Feodor-Lynen Str. 25, 81377 Munich, Germany

## Abstract

The exceptionally conserved metazoan MAB21 proteins are implicated in cell fate decisions and share considerable sequence homology with the cyclic GMP-AMP synthase. cGAS is the major innate immune sensor for cytosolic DNA and produces the second messenger 2′-5′, 3′-5′ cyclic GMP-AMP. Little is known about the structure and biochemical function of other proteins of the cGAS-MAB21 subfamily, such as MAB21L1, MAB21L2 and MAB21L3. We have determined the crystal structure of human full-length MAB21L1. Our analysis reveals high structural conservation between MAB21L1 and cGAS but also uncovers important differences. Although monomeric in solution, MAB21L1 forms a highly symmetric double-pentameric oligomer in the crystal, raising the possibility that oligomerization could be a feature of MAB21L1. In the crystal, MAB21L1 is in an inactive conformation requiring a conformational change - similar to cGAS - to develop any nucleotidyltransferase activity. Co-crystallization with NTP identified a putative ligand binding site of MAB21 proteins that corresponds to the DNA binding site of cGAS. Finally, we offer a structure-based explanation for the effects of MAB21L2 mutations in patients with eye malformations. The underlying residues participate in fold-stabilizing interaction networks and mutations destabilize the protein. In summary, we provide a first structural framework for MAB21 proteins.

The male abnormal 21 (MAB21) like proteins form a sequence related subfamily within the large and diverse family of nucleotidyltransferase (NTase) fold proteins^1^. NTases generally catalyze the transfer of a nucleoside monophosphate (NMP) from a donor nucleoside triphosphate (NTP) to an acceptor hydroxyl. A prominent member of the MAB21 like protein family is cyclic GMP-AMP synthase (cGAS), a sensor of cytosolic DNA in the innate immune system^2^,^3^. cGAS detects cytosolic DNA arising from intracellular bacteria, damaged mitochondria, DNA viruses and retroviruses and triggers a type I interferon response^4^,^5^,^6^. Upon DNA recognition, cGAS produces the second messenger, 2′-5′, 3′-5′ cyclic GMP-AMP (2′,3′-cGAMP) by two sequential NTase reactions from substrate ATP and GTP^7^,^8^.

Besides cGAS (also known as MAB21 domain-containing protein 1 - MB21D1), MAB21 like proteins in humans include MB21D2, MAB21-like protein 1 (MAB21L1), MAB21L2 and MAB21L3^1^. Although cGAS or cGAS-like activities are present in insects, vertebrates and possibly sea anemone, the MAB21 protein was first discovered in *C. elegans* as a factor important for cell fate determination^9^,^10^,^11^. Mutations in *C. elegans MAB21* cause posterior-to-anterior homeotic transformation of sensory ray 6 in the male tail. The fundamental and crucial role of MAB21 during development is emphasized by additional pleiotropic phenotypes previously described and the fact that, the null allele of *MAB21* is lethal prior to hatching. MAB21 is unusually conserved throughout species and vertebrate MAB21 proteins have almost identical primary sequences.

Despite comprehensive characterization of *MAB21* phenotypes and an emerging role in cell fate decisions, the molecular function remains unknown. In vertebrates, MAB21L1 and MAB21L2 have a 94% identical amino acid sequence and exhibit similar expression patterns^12^,^13^,^14^,^15^,^16^. In agreement with the observations in *C. elegans*, mutations of MAB21L2 result in drastic phenotypes and defects that lead to death in mid-gestation^17^,^18^,^19^. Studies on *MAB21L1* revealed relatively mild phenotypes in tissues where the expression of MAB21L1 differs from that of MAB21L2, namely in the lens and in reproductive organs^20^. MAB21L2 antagonizes the effects of BMP4 in *Xenopus* via interactions with the transcription factor SMAD1 and is therefore involved in regulation of the TGF-β pathway^21^,^22^. Moreover, MAB21L3, which only shares a sequence identity of 25%, was recently shown to be acting downstream of the Notch pathway in cell fate specifications of multiciliate cells and ionocytes^23^.

Recent current whole exome sequencing projects identified four different missense mutations in the single-exon gene *MAB21L2* in eight individuals with major eye malformations^24^. In particular, an Arg51 mutation to cysteine (R51C) led to bilateral anophtalmia, intellectual disability, and rhizomelic skeletal dysplasia^25^. A mutation of the same residue to histidine (R51H) in a large multiplex family with colobomatous microphtalmia, as well as the mutation Glu49Lys was linked to coloboma. A homozygous mutation altering a different region of the protein Arg247Gln was also associated with retinal coloboma. The underlying molecular pathology of these mutations is unclear and both protein stabilizing and destabilizing effects have been proposed^24^,^26^.

Understanding the pivotal role of MAB21 proteins during development, their pathophysiology in human disease, and their evolutionary connection to the cGAS innate immune DNA sensor is hampered because the mechanistic function, biochemical activity and interaction partners, ligands or substrates of MAB21 proteins remain elusive. Here, we provide the first crystal structure of a MAB21 protein and the structure of MAB21L1 bound to CTP. The human MAB21L1 structure exhibits a high degree of structural conservation with cGAS but features a number of differences. Our study shows that MAB21L1 would require an activating conformational change in order to possess its potential NTase activity and unveils a potential binding site for an activating ligand of this poorly understood nucleotidyltransferase. Intriguingly, the NTase contains the residues to recognize a donor NTP and active site magnesium ions, but lacks some residues that are important for NTase activity in cGAS. Finally, we provide a structure-based explanation for the effects of mutations in *MAB21L2* associated with eye malformations and show that these mutations lead to destabilization of the fold.

## Results

### Crystal Structure of Human MAB21L1

We crystallized native MAB21L1 in space group C2 with unit cell constants a = 167.7 Å, b = 177.6 Å, c = 115.6 Å, β = 126.5°. The native crystals contained 5 molecules in the asymmetric unit and diffracted to a limiting resolution of 3.05 Å. In order to determine experimental phases, we also crystallized selenomethionine substituted MAB21L1 (space group P2_1_, unit cell constants a = 122.9 Å, b = 181.0 Å, c = 131.3 Å, β = 96.5° and 10 molecules per asymmetric unit). Single anomalous diffraction (SAD) data to 3.4 Å followed by phase improvement produced an interpretable electron density map that was used to build initial models for all ten copies in the asymmetric unit using non-crystallographic symmetry restrains. This initial model was used to phase the native dataset by molecular replacement. Further model building and refinement resulted in a final atomic model with good R-factors and stereochemistry (Table 1, Materials and Methods).

**Figure 1:**
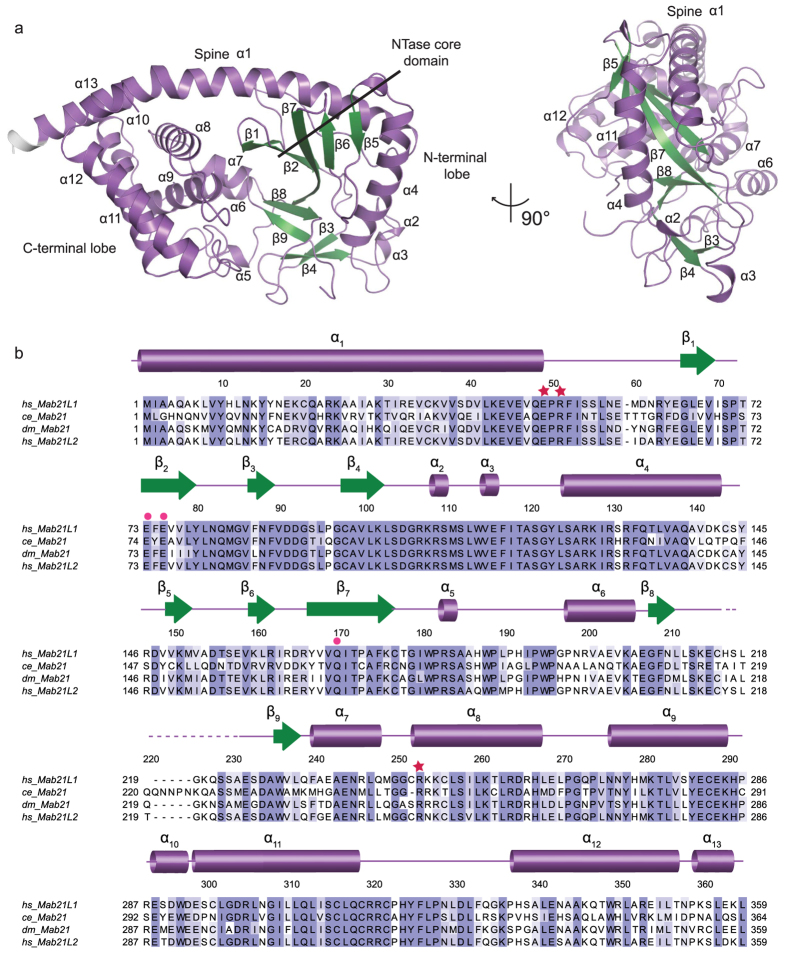
Structure of human MAB21L1 and Alignment with ceMAB21, dmMAB21 and hsMAB21L2. (**a**) Front and side views of the human MAB21L1 crystal structure. The model is shown as cartoon representation with annotated domains and secondary structure. (**b**) Sequence alignment of MAB21, MAB21L1 and MAB21L2 amino acid sequences (abbreviations: *Homo sapiens*: hs, *Drosophila melanogaster*: dm, *Caenorhabditis elegans*: ce). Darker shadings indicate higher conservation (BLOSUM62 conservation score). The respective secondary structure elements for the human homolog are shown on top of the alignment with dots marking the conserved active site residues usually involved in metal coordination. Red stars denote residues that have been identified in disease related mutations.

MAB21L1 is a two lobed, globular protein of a mixed α/β topology with approximate molecular dimensions of 64 Å × 35 Å × 48 Å. (Fig. 1a). The N-terminal lobe possesses the NTase subdomain with a five-stranded, antiparallel and highly twisted β-sheet (β1–β9) that is flanked on the surface by two long α-helices (α1/α4). The C-terminal lobe is formed by a conical four-helix bundle (α8–α13). This bi-lobal structure is stabilized by a long N-terminal ‘spine’ helix (α1), reaching across and spanning both lobes, as well as a central linker region composed of two helices (α5, α6). In general, the overall topology is highly similar to cGAS in its inactive form^8^,^27^,^28^,^29^. A notable difference with respect to cGAS is the lack of the zinc-thumb region which is important for DNA binding and dimerization^27^,^30^,^31^. Similarities and differences to cGAS and other members of the NTase fold protein family are described in more detail below.

### Oligomerization and Interaction Interfaces

**Figure 2:**
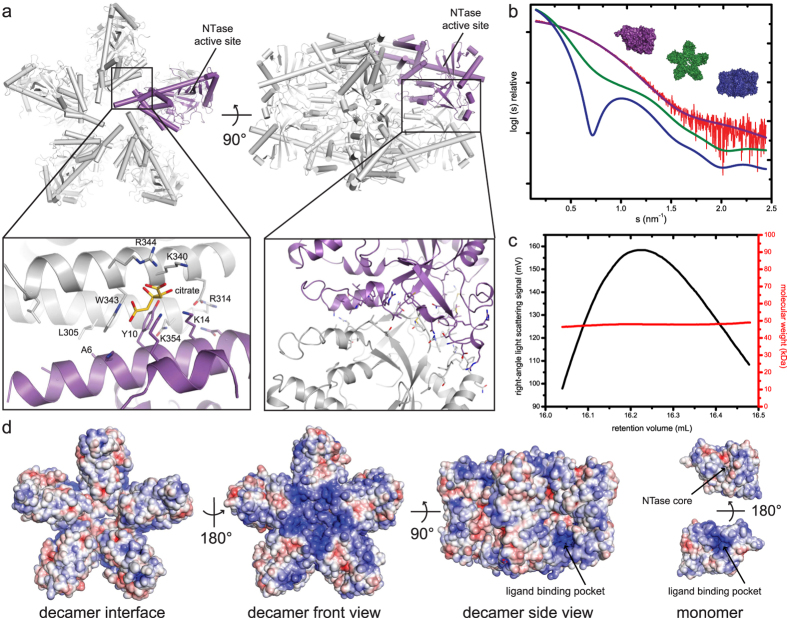
Characterization of MAB21L1 Oligomeric State in the Crystal and in Solution. (**a**) Front and side views of the MAB21L1 pentamer and decamer formation in the crystallographic asymmetric unit. One MAB21L1 monomer is colored in purple. The left inset shows a close-up of the hydrophobic interface between MAB21L1 molecules (purple, grey) in the pentameric ring. The right inset shows the interaction between two pentameric rings in the decameric assembly which is mainly composed of stacking interactions between highly conserved residues. (**b**) Measured SAXS data for MAB21L1 (red curve shows the measured scattering data) in comparison with theoretical scattering curves (calculated with CRYSOL) of monomeric, pentameric and decameric MAB21L1 assemblies (purple, green and blue curves, respectively) support that MAB21L1 is monomeric in solution. (**c**) Size-exclusion chromatography coupled static right-angle light scattering (SEC-RALS) of MAB21L1 shows a single peak with a constant molecular mass of Mw = 47 kDa. (**d**) Solvent accessible electrostatic surface representations of the MAB21L1 decamer interface, decamer (front view and side view), monomer (front view with NTase core and back view with ligand binding pocket) colored by charge (blue = 5 kT/e to red = −5 kT/e). The positively-charged ligand binding pocket is indicated with an arrow.

The crystal packing of MAB21L1 is unusual and consists of an intimate assembly of ten copies of MAB12L1 into a decamer, formed from two back-to-back stacked pentameric rings. The decamer has a D5 symmetry (one five-fold and five perpendicular two-fold symmetry axes) (Fig. 2a, Supplementary Fig. S1). Whereas the MAB21L1 protomers within the decamer are intimately packed, the decamers themselves pack less dense in the lattice and the interactions resemble crystal lattice interactions (Fig. 2a). The highly symmetric packing of MAB21L1 raises the possibility that oligomerization is part of MAB21L1’s function – similar to e.g. cGAS - and it might be worthwhile to describe the packing within the decamer in more detail. The N-terminal lobes of each protomer are partially surface accessible and located on the outside of the pentameric rings, whereas the C-terminal lobes are to a large extent buried and mediate the contacts within the pentamer. The pentamer interface is composed of hydrophobic and some polar interactions between the N-terminal helix α1 of one monomer and the C-terminal helices α11 and α12 of the neighboring molecule (Fig. 2a). Y10 stacks with W343 and interacts with a hydrophobic patch of other nearby residues (L305, I2, A6), while N20 hydrogen-bonds with R314/E336. A citrate molecule from the crystallization condition stabilizes this interface (Fig. 2a). The citrate is coordinated by residues R344 and K340 from one monomer together with K14 and K354 from the next monomer in a very basic surface patch. In total, each pentamer buries a surface area of approximately 2500 Å^2^.

The interface between the two back-to-back stacked pentamers is in the range of 5000–6000 Å^2^, i.e. the decamer contains approx. 10000 Å buried surface. The pentamer stacking is mediated by contacts between opposing lobes and the linker region, both of which are highly conserved regions of MAB21L1 and L2 (Figs 1b and 2a). Salt bridges and hydrogen bonds are formed between the linker helices α6 and α5, β3 and the loop between β2–β3 as well as between the small helices α2 and α3 from the opposing monomer. Hydrophobic interactions are formed between loops connecting β2 with β3 and loop α2–α3 (Fig. 2a). The same double-pentameric packing is observed in all three of our crystals despite of changes in space groups and unit cell constants (Supplementary Fig. S1).

In order to address the oligomeric state of MAB21L1 in solution, we performed size exclusion chromatography coupled to static light scattering (SEC-RALS) as well as small angle X-ray scattering (SAXS) experiments. Both methods show that MAB21L1 is present as a monomer in solution, at least under the assayed conditions (Fig. 2b,c). MAB21L1 elutes as a single peak with a molecular mass determined by SEC-RALS of Mw = 47 kDa, in good agreement with the theoretical mass of a monomer (Mw = 41 kDa). Molecular weight determined from the SAXS data (Mw = 41 kDa, Rg = 2.4 nm) as well as the shape of the scattering curve are only compatible with a monomeric MAB21L1 molecule in solution. In order to rule out oligomerization in the crystal due to higher salt concentrations, we performed analytical size-exclusion experiments using buffers containing different salt concentrations up to 1 M. However, MAB21L1 still remained monomeric (Supplementary Fig. S1). Although MAB21L1 is monomeric in solution, it is possible that ligand interactions may promote oligomerization, such as in the case of cGAS^30^,^31^.

**Figure 3:**
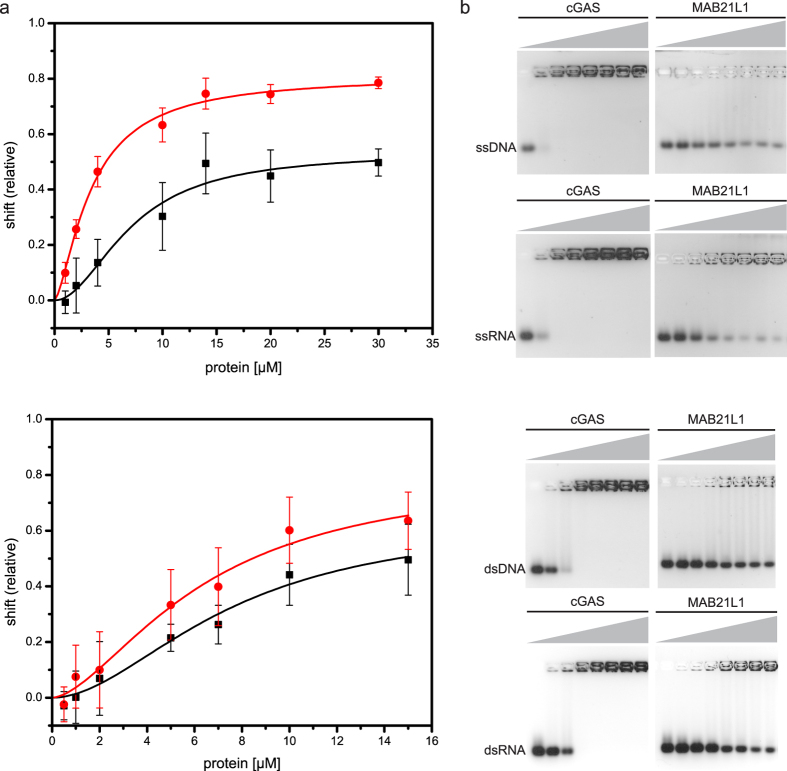
Characterization of MAB21L1’s Affinity for Oligonucleotides. (**a**) Quantified electrophoretic mobility shift assays of 1 μM 50mer dsRNA, dsDNA and 0.5 μM, ssRNA or ssDNA with MAB21L1 (protein concentrations of 0, 0.5, 1, 2, 5, 7, 10, 15 μM for binding to dsDNA/RNA and 0, 1, 2, 10, 14, 20, 30 μM for binding to ssDNA/RNA). Intensities of unbound oligonucleotides (relative shift) were plotted as triplicates against protein concentration (upper plot: ssRNA-red, ssDNA-black; lower plot: dsRNA-red, dsDNA-black). The half-maximal binding of ssRNA is at 3.3 μM protein concentration, whereas for ssDNA it is 6.7 μM, dsRNA = 5 μM and dsDNA = 6 μM, showing that MAB21L1 has a preference for ssRNA. (**b**) Corresponding gels of electro mobility shift assays of MAB21L1 in comparison to cGAS (same conditions as before). cGAS clearly has a higher affinity for generic nucleic acids than MAB21L1.

It has been proposed that MAB21L2 binds ssRNA^24^. Consistent with this, the solvent accessible electrostatic surface of the MAB21L1 monomer shows several positively-charged regions. One notable positive surface patch is a pocket at the surface adjacent to the ‘spine’ helix at the side of the double-pentamer (Fig. 2d). The corresponding region binds the DNA backbone in the case of cGAS. Another notable and quite extended patch is located around the five-fold symmetry axis of the pentamer (Fig. 2d). In order to test MAB21L1’s affinity for oligonucleotides, we performed electrophoretic mobility shift assays (EMSAs) with dsDNA/RNA as well as ssDNA/RNA (Fig. 3a,b). MAB21L1 has a preference for ssRNA in comparison to dsRNA and ss/dsDNA in good agreement with Rainger *et al*.^24^ (Fig. 3a), as can be estimated from the concentrations at half-maximal binding (ssRNA = 3.3 μM, ssDNA = 6.7 μM; dsRNA = 5 μM, dsDNA = 6 μM). The variations in the total binding of MAB21L1 to ssRNA and ssDNA are due to weak binding constants and therefore no binding curves and respective K_D_ values were calculated. However, the affinities for these generic DNA or RNA sequences are considerably lower in our hands than those for DNA binding to cGAS (Fig. 3b). We could also not detect any enzymatic NTase activity of MAB21L1 either in the absence or presence of these nucleic acids.

### Structure of MAB21L1 Bound to CTP Reveals Possible Ligand Binding Site

**Figure 4:**
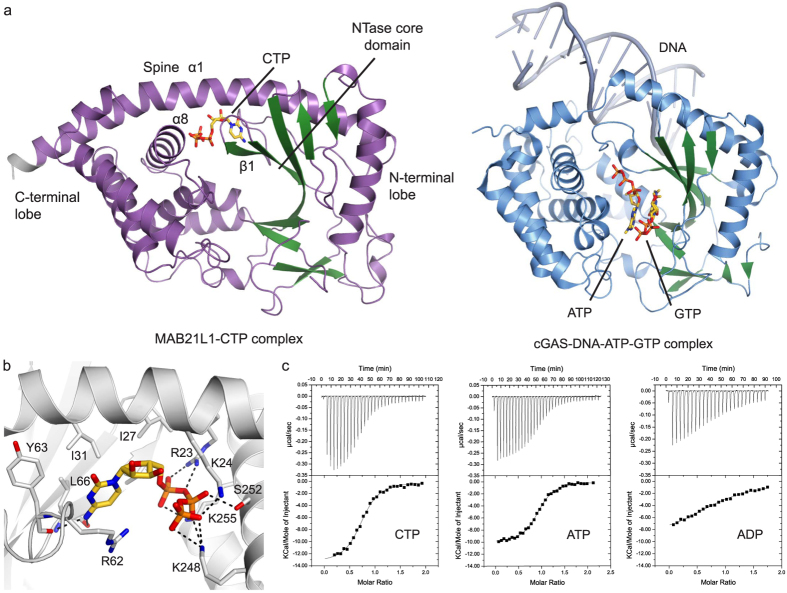
Structure of MAB21L1 in Complex with CTP. (**a**) Front views of the MAB21L1-CTP complex crystal structure (left) and the crystal structure of the cGAS^MAB21^-DNA-ATP-GTP complex (right, PDB code 4KB6). (**b**) Close-up view of the CTP binding site with the bound CTP. Key residues interacting with the CTP are depicted and annotated. The CTP is bound in a positively charged pocket via interactions with the NB-loop. (**c**) Titration of MAB21L1 with different nucleotides by ITC [K_D_(CTP) = 0.41 μM, K_D_(ATP) = 0.34 μM and K_D_(ADP) = 3.1 μM] suggest that MAB21L1 has a preference for nucleotide triphosphate coordination at this site.

In order to identify potential nucleotide substrates of MAB21L1, we performed thermal shift assays and isothermal titration calorimetry experiments using various nucleoside phosphates. We observed large increase in the melting temperature of MAB21L1 in the presence of CTP, CDP, ATP and ADP of ∆Tm = 8 °C (Supplementary Fig. S2). Other nucleotides tested, such as GTP and UTP, did not stabilize the protein and did not bind in isothermal titration calorimetry experiments (Supplementary Fig. S2). However, isothermal titration calorimetry experiments reveal strong binding of CTP, ATP and ADP with dissociation constants of K_D_(CTP) = 0.41 μM, K_D_(ATP) = 0.34 μM and K_D_(ADP) = 3.1 μM. These data indicate a preference for a triphosphate over a diphosphate (Fig. 4c).

In order to test for Mg^2+^ -binding we performed a thermal stability shift assay in the absence and presence of magnesium. Magnesium did not stabilize MAB21L1 and this might be explained by the fact that MAB21L1 is present in an inactive conformation (Supplementary Fig. S2).

To further address the nucleotide binding, we co-crystallized MAB21L1 with both ATP and CTP in the crystallization condition and determined the resulting crystal structure of MAB21L1 to 2.55 Å resolution. In this structure each protomer shows clear density for a single CTP moiety (Supplementary Fig. S3), and we did not see any density for ATP. Unexpectedly, the CTP moiety is not localized at the NTase active site, but rather bound at the positively-charged pocket on the ‘platform’ side of the molecule adjacent to the spine helix (Fig. 4a). The pocket coincides with a positively-charged surface patch on the ‘side’ of the double-pentamer (Fig. 2d). We denote this pocket ‘ligand binding pocket’. CTP is well coordinated in a partially hydrophobic, partially positively-charged pocket. In the loop connecting α1-β1, R62 stacks with the cytosine base in a planar conformation (Fig. 4b). This loop is better resolved in the MAB21L1:CTP structure, compared to *apo* MAB21L1, arguing for some sort of induced fit. Further contacts of MAB21L1 with CTP include hydrophobic interactions between I31, I27, V68 and A28 and carbons of the base and sugar. Backbone interaction between L66 and Y63 and the amino group at C4 of the cytosine explain the preference of MAB21L1 for CTP and ATP, compared to UTP and GTP where no binding affinity was detected. The negative charge of the phosphate groups of the nucleotide is well and specifically coordinated by residues from α1 and α8. The α-phosphate is coordinated by K255 and R23, the β-phosphate builds salt bridges with K255 and S252 and R23, while the γ-phosphate is held in place by K24 and K248. During model building of the MAB21L1 structure with no nucleotides added, we observed an additional electron density not associated with the polypeptide chain in the ligand binding pocket (Supplementary Fig. S3). However, we were not able to identify by structural or mass spectrometric methods the nature of this low molecular weight ligand, which may be a buffer component or have co-purified with the protein from *E. coli*.

In summary, CTP binding did not obviously affect the overall conformation of MAB21L1 and the superposition of *apo* MAB21L1 (crystallized without added nucleotides) and MAB21L1 in complex with CTP reveals no major conformational changes (RMSD 0.94 Å) (Supplementary Fig. S3). Thus, it is unlikely that CTP is the physiological ligand that can induce a potential activating conformational change (see below).

### Comparison of MAB21L1 and cGAS Active Sites

**Figure 5:**
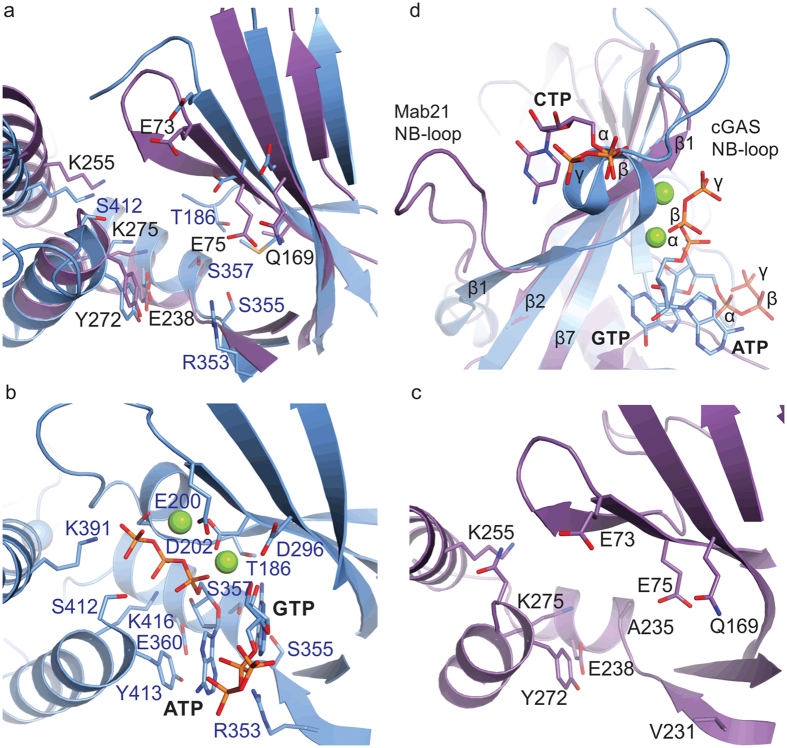
Superposition of MAB21L1 Active Site with Structurally Conserved cGAS. (**a**) Close-up views of the NTase active sites of human MAB21L1 (purple) superposed with porcine cGAS (blue, PDB code 4JLX). (**b**) The corresponding part of the porcine cGAS-DNA-ATP-GTP (blue, PDB code 4KB6) and (**c**) human MAB21L1 structure in the same orientation. Residues responsible for magnesium ion coordination in the cGAS-DNA-ATP-GTP structure (PDB code 4KB6) Q200 and N202 are mutated in the figure to E200 and D202, respectively, and a second magnesium ion was added to the catalytic site of cGAS for reason of clarity. Selected residues of MAB21L1, which are conserved to cGAS residues implicated in catalysis, are annotated. (**d**) Close-up view of nucleotide binding sites in the cGAS-DNA-ATP-GTP and MAB21L1-CTP complexes. CTP binding to MAB21L1 prevents the NB-loop rearrangements required for activation and therefore access to the catalytic pocket.

To reveal differences and similarities between MAB21L1 and cGAS in the NTase active sites, we superposed the structures of N-terminally truncated porcine *apo* cGAS (RMSD 2.2 Å, PDB code 4JLX) and porcine cGAS^MAB21^-DNA-ATP-GTP complex (RMSD 2.8 Å, PDB code 4KB6) with the structure of the MAB21L1:CTP complex (Fig. 5). MAB21L1 shows a high degree of structural conservation to cGAS and the conserved MAB21-domain fold strongly resembles other human nucleotidyltransferases, such as OAS1, and the bacterial DncV protein (Supplementary Fig. S4). In general, many active site residues located on the α-helical C-terminal lobe – responsible for binding of the ‘donor’ nucleotide in cGAS and other NTases, such as OAS1 - are highly conserved (Supplementary Fig. S4). For instance, Y272 in MAB21L1 corresponds to Y413 in *ss*cGAS and to Y436 to *hs*cGAS, where it stacks with the adenine base (Fig. 5). Further conserved residues essential for correctly positioning of ATP in the active site of cGAS are *ss*cGAS E360/*hs*cGAS E383 (MAB21L1 E238), *ss*cGAS K416/*hs*cGAS K439 (MAB21L1 K275) and *ss*cGAS K391/*hs*cGAS K414 (MAB21L1 K255). Thus, at least from sequence conservation, the structure of MAB21L1 suggests that MAB21-like proteins may bind an NTP at the NTase donor position.

The N-terminal lobe binds the Mg^2+^ -triphosphate group of the donor nucleotide and specifically recognizes and orients the ‘acceptor’ nucleotide GTP in cGAS. Consequently, the Watson-crick and Hoogsteen edges of the guanine base are within hydrogen bonding distance of the residues *ss*cGAS T186, S355, R353 and S357 (*hs*cGAS T211, S378, R376 and S380) respectively. Whereas the donor site is partially conserved between MAB21L1 and cGAS, these residues, which confer cGAS specificity for the guanine base, cannot be assigned, such as *ss*cGAS T186 on the NB-loop, or are missing in MAB21L1 (A235, V231 and Q233, respectively).

A yet unanswered question is whether MAB21 proteins have any NTase activity, like e.g. cGAS or OAS. The catalytic triad on lobe I, which coordinates the two Mg^2+^ ions and polarizes the acceptor hydroxyl group in the active site of cGAS is only partially conserved in MAB21 proteins. While the presence of the Mg^2+^ ion coordinating MAB21L1 E73 and E75 (*ss*cGAS: E200 and D202/*hs*cGAS E225 and E227) suggest the existence of a functional Mg^2+^ and perhaps triphosphate coordinating site, MAB21L1 has a glutamine (Q169) at the position of *ss*cGAS D296 (*hs*cGAS D319). The carboxylate of D296 coordinates a second Mg^2+^ and helps to polarize the acceptor hydroxyl. Alanine mutations in either of the three residues or a double E200Q, D202N mutation in sscGAS lead to inactive proteins *in vitro* or in cellular assays^8^. It is unclear whether a more conserved asparagine at position hscGAS D319, similar to the Q169 of MAB21L1 still allows NTase activity.

However, in case MAB21L1 is an NTase, it would likely require a conformational change for activation. cGAS and OAS undergo conformational changes upon activation and especially part of the β1–β2 hairpin becomes a α-helical turn, which then constitutes the so called NB-loop^32^. In structures of inactive cGAS or OAS1 the loop connecting β1 and β2 is disordered in the crystal structures. Although the equivalent region of MAB21L1 is ordered in our crystal structure, it notably does not adopt the α-helical NB-loop conformation. CTP binding to MAB21L1 appears to stabilize the current, presumably inactive, conformation (Fig. 5d).

In summary, the structure of MAB21L1 exhibits similarities and differences with the structures of cGAS and OAS1, explaining why we currently fail to see NTase activity. Overall, the active site conformation observed in the crystal structure is inconsistent with catalytic activity. Residues that bind the donor NTP in the active site of NTases as well as coordinate the di-metal center are to a considerable extent conserved to cGAS, OAS proteins and other 3′-specific nucleotidyltransferases, such as poly(A) polymerase (PAP) and CCA-adding enzyme (CCA)^32^.

### Human Missense Mutations of MAB21L2 Mapped on MAB21L1 Structure are Responsible for Protein Stability

A whole exome sequencing project revealed four different MAB21L2 missense mutations in patients with ocular coloboma. Out of 38 candidate genes for eye malformations, MAB21L2 was the only one altered by mutations. Previous studies attempted to explain the drastic phenotype of these mutations based on alignments of MAB21L2 with cGAS^24^. However, only the mutation R247Q, located in the C-terminal lobe on α8, is structurally conserved in cGAS and even OAS1 (Supplementary Fig. S4) and previous work disagreed whether the patient derived mutations increased or decreased protein stability^24^,^26^.

**Figure 6:**
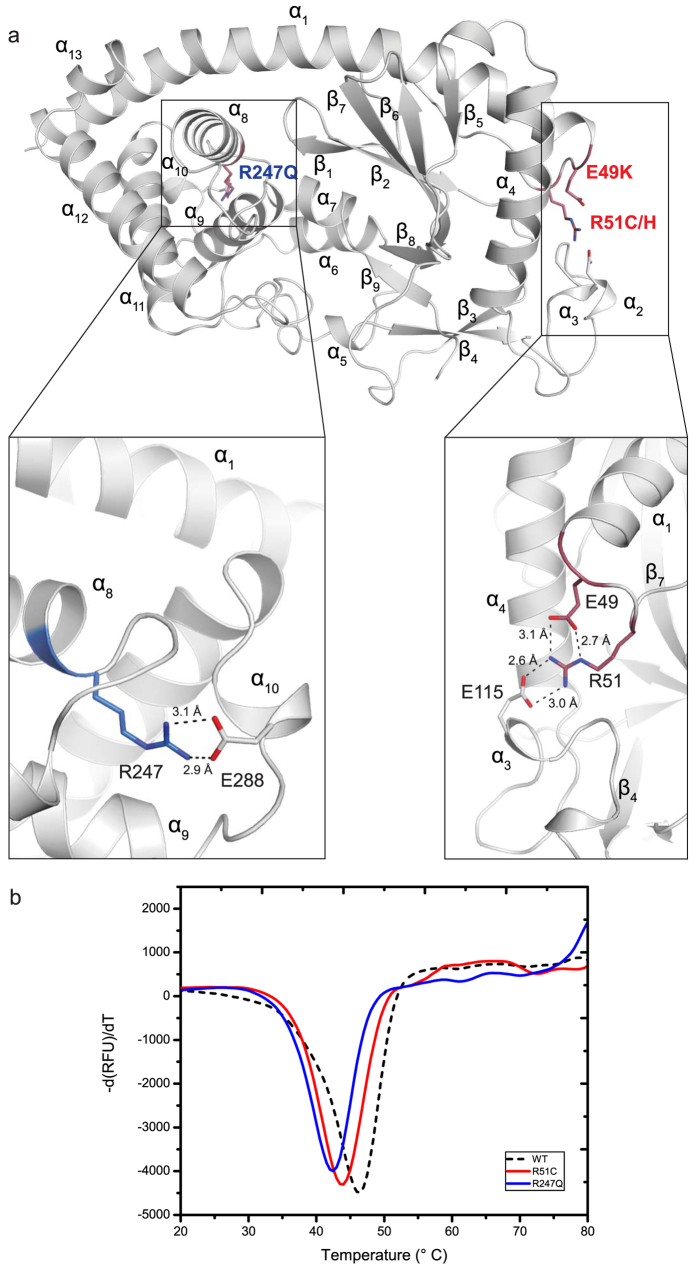
Patient Mutations on MAB21L2 Affect Protein Stability. (**a**) Amino acids of MAB21L2 mutated in patients with eye malformations are depicted in blue (R247Q) and red (E49K, R51C/H) in the MAB21L1 structure. The insets show close-up views of the salt bridges formed by the mutated residues. R247 forms a salt bridge with E288, while E49 and R51 build a network of salt bridges with E115. Mutation of these stabilizing interactions will affect the correct MAB21L1 fold. (**b**) Thermal shift assay derivative melt curve plots of MAB21L1 (black), MAB21L1 R51C (red) and MAB21L1 R247Q (blue). The disease related mutations in MAB21L1 all show a decreased melting temperature supporting the idea that the mutations destabilize the protein.

All of the described amino acids mutated in MAB21L2 are conserved in MAB21L1 (Fig. 1b), so our crystal structure offers a good model to reveal the molecular basis for this disease. We therefore mapped the mutations R51C/H, E49K and R247Q on our MAB21L1 structure, in order to shed light on the possible effects on the protein stability (Fig. 6a). In our MAB21L1 structure R247 forms a salt bridge with E288 on the α10-turn. This salt bridge is disrupted by the mutation of R247 to glutamine and consequently may lead to decreased stability of the protein. Residues E49 and R51 (Mutated to K and C/H, respectively) are located on the beginning of loop α1-β1. They are unique to MAB21 proteins and are highly conserved (Fig. 1b). Based on our MAB21L1 structure, E49 and R51 participate in a network of salt bridges with E115 (Fig. 6a). E49 and R51 stabilize the large loop between β4 and α4 by anchoring the loop via interactions to E115. This loop and E115 are a highly conserved element in MAB21L1 and L2, indicating that destabilization caused by the mutations of E49 to lysine and R51 to cysteine or histidine inactivate MAB21L2.

To experimentally address the effects of the mutations on the protein stability we performed comparative fluorescence thermal shift assays. The thermal stability of MAB21L1 was tested in comparison to MAB21L1 R247Q and MAB21L1 R51C. MAB21L1 R51C and MAB21L1 R247Q showed significantly decreased melting temperatures, which indicates a decreased stability of the mutated protein (Fig. 6b). The mutant MAB21L1 R247Q showed an even more drastic decrease in melting point stability loss as R51C. The fact that R247Q is also conserved in cGAS (*ss*cGAS R383 and *hs*cGAS R406) suggests that this residue plays a more fundamental role in protein stability (Supplementary Fig. S4). In summary, we conclude that the MAB21L2 mutations associated with ocular coloboma lead to a protein with reduced stability because critical fold-stabilizing bonding networks are disrupted.

## Discussion

MAB21 family proteins are implicated in cell fate decision processes in metazoans. Whereas MAB21 is required for pattern formation of the male tail in nematodes, MAB21 is sufficient and required for development of the dorso-ventral axis in vertebrates^10^,^33^,^34^. MAB21 proteins act downstream of the TGF-β signaling pathway by antagonizing the effect of BMP4 in *Xenopus* embryo ventralization and in *C. elegans* MAB21 is negatively regulated by cet-1 (vertebrate BMP4)^22^. Direct interactions of MAB21L2 with SMAD1, a nuclear transducer of the TGF-β pathway, suggest that MAB21L1 could be a transcriptional repressor^21^. Several studies in the model organisms *Xenopus*, zebrafish and mouse revealed that both genes have similar expression patterns in the developing eye, mid- and hindbrain, neural tube and branchial arches^13^,^14^,^15^,^35^. In particular, recent studies in zebrafish detected *MAB21L2* transcripts in the ciliary marginal zone of the retina. Similarly, expression of *MAB21L3* in *Xenopus* was specific for multiciliate cells and ionocytes associating MAB21 proteins with cell fate specification of cilia^23^,^26^.

The biochemical activity and physiological role of MAB21 proteins remain to be established. As with many other NTases there are three ligand or substrate molecules to be considered, which make identification of the biochemical activity and biological function of these types of molecules challenging. NTases transfer a NMP from a donor NTP onto a hydroxyl group of an acceptor molecule. A third molecule required for development of catalytic activity is a potential activating ligand. In the case of cGAS, these molecules are ATP, GTP and double-stranded DNA. In the case of MAB21 proteins, neither the substrates of a NTase reaction nor a potential activating ligand have been described, if such molecules exist at all. Our structural studies show that MAB21L1 resembles cGAS in the unliganded inhibited state and show that any NTase activity would require a conformational change similar to that DNA induces in cGAS^8^,^27^. It is of course possible that MAB21 proteins exert their functions via a predominantly structural role, e.g. by binding other proteins or nucleic acids such as mRNAs or micro-RNAs, and do not possess any catalytic activity at all. For instance, it has previously been proposed that MAB21L2 interacts with mRNA^24^. We also see some preferential but moderate binding to generic ssRNA with MAB21L1 and the positive electrostatic surface potential would be consistent with nucleic acid binding. Generic nucleic acids bind, however, with considerably less affinity to MAB21L1 than to cGAS^27^. This reduced affinity suggests that MAB21 binds perhaps a specific sequence or structure, if any. Of note in this context, potential functional link to micro-RNAs in the case of MAB21L3 has been proposed, although there are no experimental data for a functional or physical interaction at this point^23^. Apart from nucleic acids, it is also possible that MAB21 proteins interact with other protein partners such as the SMAD proteins^21^.

MAB21 proteins could also confer their function through a catalytic NTase activity. As stated above, MAB21L1 has an inactive conformation in the crystal structure, but a cGAS-like conformational change could in principle form a proper active site. If such a hypothetical conformational change takes place, our structure shows that residues typically associated with donor NTP binding are conserved, in particular at the C-terminal lobe 2. These include Y272, which stacks with the base, and E238, which binds to the sugar OH groups. Finally, donor NTP binding also requires the catalytic triad carboxylates on lobe I, which coordinate together with the triphosphate moiety the two active site magnesium ions. However, residues coordinating the acceptor nucleotide GTP in cGAS are not found in MAB21L1 and residues located on the NB-loop cannot be assigned in our conformation, due to the lack of a MAB21L1 structure in an active conformation. Furthermore, in MAB21L1 and L2, two out of the three catalytic triad residues are conserved (E73, E75), whereas the third triad residue (Q169) is altered. In the case of cGAS the aspartate equivalent to Q169 (*hs*cGAS D319) coordinates a second active site Mg^2+^ and could help to polarize the attacking acceptor OH. A glutamine at this position could still coordinate this magnesium ion, but may result in a reduced but perhaps not entirely abolished activity. As such, we do not want to rule out the possibility that MAB21 proteins still possess NTase activity.

An unexpected observation was that CTP was not bound at the NTase active site, even though it binds to MAB21L1 with 0.41 μM affinity and leads to a substantial increase in thermal stability. In previous studies by ourselves and other groups with related enzymes (cGAS, OAS, MiD51), co-crystallization with NTPs or soaking of crystals with NTPs resulted in binding of the NTPs at the NTase active site, although the NTPs were often bound in a catalytically inactive conformation and not sufficient to switch the enzyme from an inactive to an active conformation^27^,^32^,^36^,^37^. In our structure, CTP is bound to a site that recognizes activating ligands in the case of related NTases (cGAS, OAS1), and it is tempting to speculate that this site is a ligand-binding site of MAB21 proteins as well. Obviously CTP did not switch the protein into an active conformation, so CTP is unlikely a physiological activating ligand. It is possible however, that CTP mimics part of a physiological ligand. It should be noted that the sugar and base moieties of CTP are bound by the NB-loop in its inactive conformation and thus would argue against an activating structural switch in MAB21, while the triphosphate binds into a highly positively-charged pocket that may not be affected by a conformational switch. The fact that the structure of MAB21L1 without any nucleotides added shows density for an unidentified molecule in the ligand binding pocket may support the suggestion that this positively-charged site acts as a binding site for an acidic ligand. The explicit preference of MAB21L1 for a nucleoside triphosphate (over a diphosphate) at this site suggests that MAB21L1 could bind, for instance, the 5′ triphosphate of an RNA such as mRNA or precursor miRNA. This positively-charged pocket in MAB21L1 could act as an activation site but also as an inactivation site, such as the equivalent site on the bacterial nucleotidyltransferase DncV that binds 5MTHFGLU2^38^. In the folate-bound conformation of DncV residues Y117 and Q116 sterically hinder binding of nucleotides in the active site.

In our structure, MAB21L1 packs into a decamer with D5 symmetry. This highly symmetric packing is quite unusual for generic crystal lattice interactions. Although we cannot rule out that this packing is purely a result of the high concentrations in the crystal, it may have a biological function, even though it was not detected *in vitro* in solution. cGAS, for instance, was shown to cooperatively bind dsDNA through two distinct interaction sites, which induced oligomerization required for activation^30^,^31^. The interaction interfaces between protomers in the double pentamer are rather small and distinct from those of the oligomers of related proteins, such as cGAS and MiD51^30^,^36^. The interface area between two pentameric rings, however, is substantial and mediated by a highly conserved region in MAB21L1 and L2 comprising amino acids G96-Q138 (Fig. 1b). The same sequence was shown to be involved in protein-protein interactions in related MAB21 domain proteins. Drp1 recruitment to MiD51 was abolished when a single amino acid was mutated in this region^36^. In addition, residues differing between the 94% identical MAB21L1 and MAB21L2 proteins do not affect the predicted oligomer interface. The extreme surface conservation of MAB21 proteins throughout species further argues for potential oligomerization and/ or interaction partner binding features. Therefore, the oligomerization of MAB21 could be of biological importance and should be considered in future studies.

Finally, our structure allows a molecular interpretation of the MAB21L2 mutations in ocular coloboma since the underlying residues and their interaction partners are conserved between L1 and L2. Interestingly, we find that the mutated residues are not located on the surface but rather stabilize the structure of MAB21L1/L2. Consistently, the mutations reduce the thermal stability of MAB21L1 *in vitro*, suggesting that the disease is caused by destabilized MAB21L2. Of note, the mutations E49K and R51C/H help fold a highly conserved area in MAB21L1/L2 that in our structure mediates the pentamer-pentamer interactions. In MiD51 the same loop is responsible for protein-protein interactions^36^. Therefore, the phenotype caused by mutations E49K and R51C/H could be due to disruption of possible protein-protein interactions with a yet unknown partner, that might be required for MAB21L1 to fulfill its function. Furthermore, these mutations were previously shown to disrupt MAB21L2 ability to bind ssRNA, consistent with the locations of the affected residues close to the RNA binding cleft in OAS1^24^.

In line with the mutations observed for MAB21L2, a recent study reports severe effects of a the Cys246Leufs*18 frameshift mutation in MAB21L1^39^. This mutation leads to a potentially disrupted structural integrity of the whole alpha helical arrangement in the C-terminal lobe, also affecting the mapped intramolecular interaction R247:E288 in MAB21L2 mutation R247Q. The fact that the MAB21L1 mutation causes an additional phenotype compared to MAB21L2 argues for an important role of both proteins in the organism.

In summary, we provide a first structural framework for the highly conserved MAB21 family of nucleotidyltransferase fold proteins.

## Methods

### Protein Expression and Purification

The full length human MAB21L1 gene, purchased from GENEART with codon optimization for expression in *E. coli*, was cloned into a modified pET21 vector with an N-terminal His-MBP-tag. Site-directed mutagenesis was performed using the Quikchange (Stratagene) protocol with the respective primers. Expression was performed in *E. coli* BL21 Rosetta (DE3) and B834 (DE3) strains for native and selenomethionine labeled proteins, respectively. Cells were induced with 0.1 mM IPTG after reaching an OD_600_ 0.5–0.6 and proteins were expressed at 18 °C for 18 h. Harvested cells were resuspended in lysis buffer (50 mM Tris, 300 mM NaCl, 25 mM imidazole, 2 mM β-mercaptoethanol, 5% v/v glycerol) and disrupted by sonication. MAB21L1 was purified by nickel-affinity chromatography and the His-MBP-tag was subsequently removed by TEV-protease cleavage (ratio 1:30) at 4 °C over night in buffer A (30 mM Tris-HCl, 100 mM NaCl, 2 mM DTT, pH 7.0). The protein was then further purified by cation exchange chromatography step using a HiTrap SP HP column (GE Healthcare) at pH 7.0. The protein was eluted with a linear gradient up to 50% buffer B (30 mM Tris, 1 M NaCl, 2 mM DTT, pH 7.0). Fractions containing MAB21L1 were pooled and loaded on a Superdex 75 size exclusion chromatography column (GE Healthcare) using 20 mM Tris-HCl pH 7.5, 150 mM NaCl and 2 mM DTT as running buffer. MAB21L1 fractions were concentrated (2–3 mg/ml) before being flash-frozen in liquid nitrogen for storage at −80 °C. MAB21L1 mutants were expressed and purified accordingly. The final crystallization buffer for selenomethionine derivatized protein contained 2 mM TCEP (Tris(2-carboxyethyl) phosphine).

### Crystallization of MAB21L1

Co-crystallization of MAB21L1 with a mixture of CTP/ATP (5 mM final concentration) was performed by hanging drop vapor diffusion in 0.1 M MES pH 5.5 and 1 M tri-sodium citrate. 2 μL protein/nucleotide mix were added to 1 μL reservoir solution from a total reservoir volume of 250 μL in the well. Native crystals grew for one week at 20 °C before being mounted on a Free Mounting System (FMS, Proteros Biostructures GmbH). Liquid surrounding the crystal was removed in the humidified gas stream with a glass capillary. TMAO (trimethylamine oxide) was supplied as a cryo-protectant to the protein crystal with the PicoDropper during a humidity gradient. The crystal was then flash-frozen by the quick rotational replacement of the humidity nozzle with the cryo nozzle and finally stored in liquid nitrogen (for more details see also^40^). Selenomethionine derivatized protein crystals grew in 0.2 M Tri-potassium citrate pH 7.8 and 16% PEG 3350 and were cryo protected by soaking in reservoir solution containing 30% (v/v) glycerol prior to flash freezing.

### Data Collection and Structure Determination

X-ray diffraction data were collected at beamline SLS X06SA (Swiss Light Source, Villigen, Switzerland) and PETRAIII beamline P13 (EMBL/DESY, Hamburg, Germany). Diffraction data were processed using XDS and XSCALE^41^. We determined the structure of MAB21L1 using a high-redundant single anomalous diffraction (SAD) dataset measured at the Se-peak wavelength. Three finely-sliced datasets of translations on one needle-shaped crystal were merged (10800 × 0.1° frames) to provide enough phase information. The self-rotation function calculated with MOLREP^42^ of the CCP4 package^43^ suggested the presence of an additional five-fold symmetry axis and in combination with the Matthews’ coefficient we assumed either 10 or 15 copies. Phasing and density modification using the PHENIX suite^44^ located 70 Se sites for the 10 copies of MAB21L1 in the ASU leading to an initial low resolution map at 3.4 Å. This map allowed partial automatic model building using BUCCANEER^45^ followed by manual model building in COOT^46^ and alternate refinement steps in PHENIX. The initial starting model could be used to phase the two better diffracting native crystals using PHASER^47^, leading to readily interpretable electron density. Extensive manual model building in COOT and further iterative refinements with PHENIX resulted in a final model with reasonable statistics. Data collections and refinement statistics are listed in Table 1. Figures were created with PyMOL^48^.

### Thermal Shift Assay

The thermal stability of MAB21L1 in presence of different ligands was analyzed by fluorescence thermal shift assays. 20 μM MAB21L1 was incubated with 5 mM of the respective ligand in 25 mM Tris pH 7.5, 150 mM NaCl, 10 mM MgCl_2_. After addition of SYPRO Orange (final concentration of 5×, Invitrogen) the fluorescence signal was detected during a temperature gradient between 5 °C to 100 °C at a rate of 0.5 K/30 s with one scan per 0.5 K in a real-time thermal cycler using the FRET mode (CFX96 touch/Biorad).

### Isothermal Titration Calorimetry

Isothermal Titration Calorimetry was carried out at 23 °C using a VP-ITC Microcal calorimeter (Microcal, GE Healthcare). Proteins were dialyzed overnight against 20 mM HEPES pH 8.0, 150 mM NaCl, 1 mM TCEP and nucleotides were resuspended in dialysis buffer. 1.44 mL of protein in the cell with concentrations of 5–10 μM were titrated with nucleotides with 10× higher concentrations. 8 μL nucleotides was injected 25–30 times with 3.5 min intervals between injections. A background curve for each titration consisting of the titration of the nucleotide into buffer without protein was subtracted to account for heat dilution. The ITC data were analyzed using the Origin version 7 software package of the ITC instrument (Microcal).

### Analytical Size-exclusion chromatography

Analytical size-exclusion chromatography with MAB21L1 was performed with a Superdex 200 increase 10/300 (GE Healthcare) in 20 mM HEPES pH 8.0, 2 mM DTT with varying concentrations of NaCl (150 mM, 500 mM, 750 mM and 1 M).

### Size-exclusion chromatography coupled static light scattering

Size-exclusion coupled static right-angle light scattering (SEC-RALS) was performed using an AEKTAmicro system (GE Healthcare Life Sciences) equipped with a refractive index detector (VE 3580) and a TD270- RALS –device (Viscotek/Malvern Instruments) with a Superdex 200 10/300 size-exclusion column (GE Healthcare). BSA (66 kDa) was used as standard protein for system calibration. Analysis of data was performed with Software OmniSEC (Viscotek/Malvern Instruments).

### Small-Angle X-ray Scattering

Small-angle X-ray scattering (SAXS) experiments were performed at PETRAIII beamline P12 (EMBL/DESY, Hamburg, Germany). Protein samples were purified by size-exclusion chromatography (SEC) and centrifuged prior to the measurements. All samples were monodisperse as judged from the SEC- chromatograms and dynamic light-scattering size distributions. The scattering of the SEC running buffer (20 mM Tris-HCl pH 7.5, 150 mM NaCl and 2 mM DTT) was used for buffer correction of the MAB21L1 samples which were measured at different concentrations (1.3–6.5 mg/mL). The samples did not show signs of radiation damage and the scattering data were processed in the ATSAS suite^49^ as e.g. described in^50^. Guinier-plot (ln I(s) vs s^2^) analysis did not show signs of aggregation of the sample and provided a radius of gyration for MAB21L1 of Rg = 2.4 nm. By extrapolation to zero angle scattering intensity (I_0_) in the Guinier region s*R_g_ < 1.3 the molecular weight of MAB21L1 in solution was determined Mw(I_0_) = 49.8 kDa using BSA (66 kDa) as a I_0_-reference. This is in good agreement with the molecular weight determined from the Porod volume Mw(porod) = 41 kDa. Theoretical scattering curves of crystallographic coordinates were calculated using CRYSOL^51^.

### Electrophoretic Mobility Shift  Assays

1 μM of 50mer ds/ssDNA or ds/ssRNA (5′-GGATACGTAACAACGCTTATGCATCGCCCCGCTACATCCCTGAGCTGAC-3′), RNA same sequence as DNA) was incubated with the indicated increasing concentration of purified protein for 30 min on ice. As reaction buffer 20 mM Tris pH 8.0 and 200 mM NaCl was used. Samples were separated by a 1% agarose gel supplemented with Gel-Red (Biotium) for staining as suggested by the manufacturer instructions.

## Additional Information

**Accession codes:** MAB21L1 coordinates and structure factors have been deposited in the Protein Data Bank with the accession codes 5EOG (*apo*-form) and 5EOM (CTP-bound), respectively.

**How to cite this article**: de Oliveira Mann, C. C. *et al.* Structural and biochemical characterization of the cell fate determining nucleotidyltransferase fold protein MAB21L1. *Sci. Rep.* **6**, 27498; doi: 10.1038/srep27498 (2016).

## Supplementary Material

Supplementary Information

## Figures and Tables

**Table 1:** Data collection and refinement statistics.

Data collection	Mab21L1	Mab21L1-CTP	SeMet-Mab21L1
Space group	C2	P2_1_	P2_1_
Cell dimensions			
*a, b, c* (Å)	167.1, 177.0, 115.1	115.0, 177.8, 134.9	122.9, 181.0, 131.2
α, β, γ (°)	90, 126.5, 90	90, 97.6, 90	90, 96.5, 90
Wavelength	1.00000	1.00000	0.97898
Resolution (Å)	50-3.05 (3.13-3.05)	50-2.55 (2.62-2.55)	50-3.4 (3.49-3.4)
R_sym_	4.4 (115.7)	7.5 (151)	22 (112)
I/σI	22.5 (2.1)	21.7 (2.35)	8.75 (1.88)
Completeness (%)	98.8 (98.1)	99.8 (99.7)	99.2 (98.7)
Redundancy	7.0 (7.2)	13.9 (14.0)	8.9 (8.5)
Refinement			
Resolution (Å)	50-3.05	50-2.55	
No. reflections	50741	174180	
R_work_/R_free_	22.3/27.9	19.9/22.7	
No. of atoms (total)	13186	28115	
Protein	13117	27413	
Ligand/ion	65	440	
Water	4	262	
B-factors			
Protein	149	93	
Ligand/ion	163	109	
Water	126	77	
R.m.s. deviations			
Bond lengths (Å)	0.009	0.003	
Bond angles (°)	1.201	0.765	
Ramachandran statistics			
favoured (%)	96.3	96.8	
allowed (%)	3.3	3.0	
disallowed (%)	0.4	0.2	
PDB code	5EOG	5EOM	

Highest resolution shell is shown in parenthesis.
