# Supplementary material for: Structural and biochemical characterization of the cell fate determining nucleotidyltransferase fold protein MAB21L1
Source: Sci Rep. 2016 Jun 8;6:27498. doi: 10.1038/srep27498 (PMC4897736; doi:10.1038/srep27498)
Supplement: Supplementary Information [file srep27498-s1.pdf]

Supplementary data

Structural and biochemical characterization of the cell fate determining nucleotidyltransferase fold protein MAB21L1

Carina C. de Oliveira Mann[1], Reiner Kiefersauer[2], Gregor Witte[1*], and Karl-Peter Hopfner[1,3*]

[1]Ludwig-Maximilians-Universität München, Gene Center and Dept. of Biochemistry, Feodor-Lynen-Str. 25, 81377 Munich, Germany

[2]Proteros Biostructures GmbH, Bunsenstraße 7a, 82152 Martinsried, Germany

[3]Center for Integrated Protein Science (CIPSM), Ludwig-Maximilians Universität München, Feodor-Lynen Str. 25, 81377 Munich, Germany

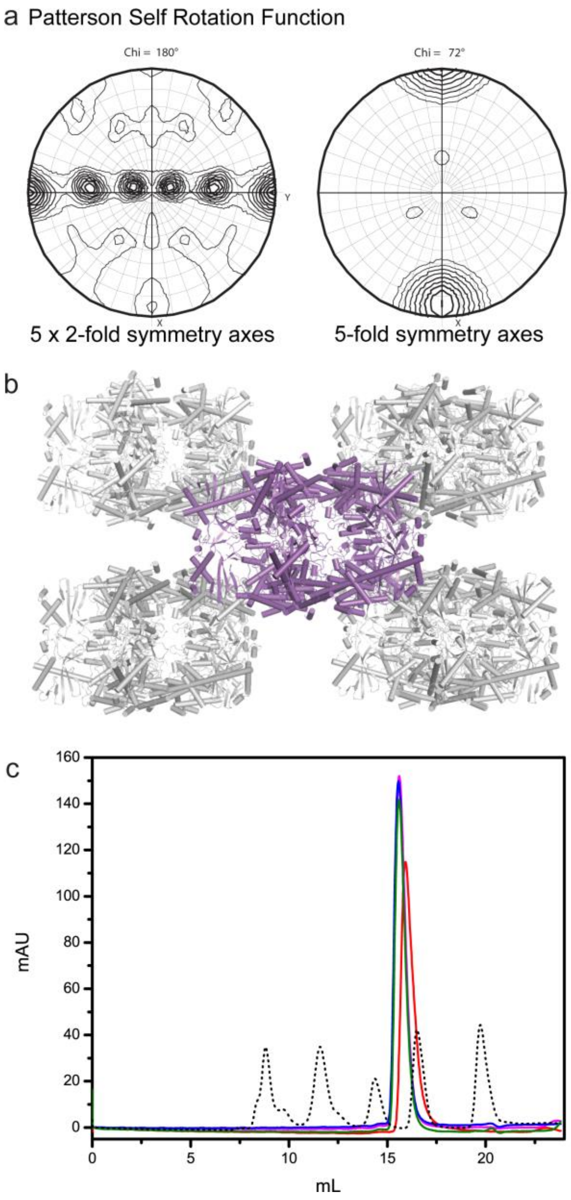

Supplementary figure S1: Patterson self-rotation function, crystal packing and analytical SEC
(a) The Patterson self-rotation function shows five two-fold non-crystallographic symmetry axes at chi=180° and a single five-fold non-crystallographic symmetry axis at chi=72°. (b) MAB21L1 crystal packing forms the decameric assembly (double-pentameric rings) observed in all three crystals, despite the different space groups and unit cell constants. (c) Size-exclusion chromatography of MAB21L1 performed with buffer containing varying NaCl concentrations (150 mM NaCl – red, 500 mM NaCl - purple, 750 mM NaCl - blue, 1M NaCl - green). Purple, blue and green chromatograms overlap. The retention volume of MAB21L1 corresponds to a monomer compared to the gel filtration standard (670 kDa, 158 kDa, 44 kDa, 17 kDa and 1.3 kDa).

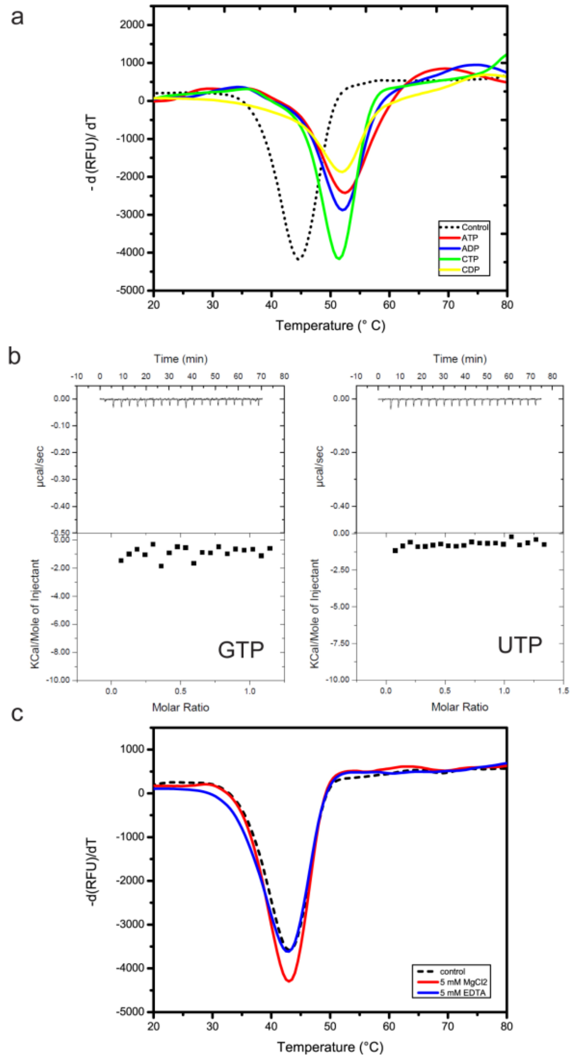

Supplementary figure S2: Fluorescence thermal shift assays and ITC of MAB21L1 with different NTPs and Mg^{2+}

(a) Fluorescence thermal shift assay derivative melt curve plots of MAB21L1 (black, dots), MAB21L1 with 5 mM ATP (red), ADP (blue), CTP (green) and CDP (yellow). (b) Titration of MAB21L1 with GTP and UTP by ITC show no binding to MAB21L1. (c) Fluorescence thermal shift assay derivative melting curve plots of MAB21L1 (black, dots), MAB21L1 in presence of 5 mM $MgCl_2$ (red) and MAB21L1 with 5 mM EDTA (blue).

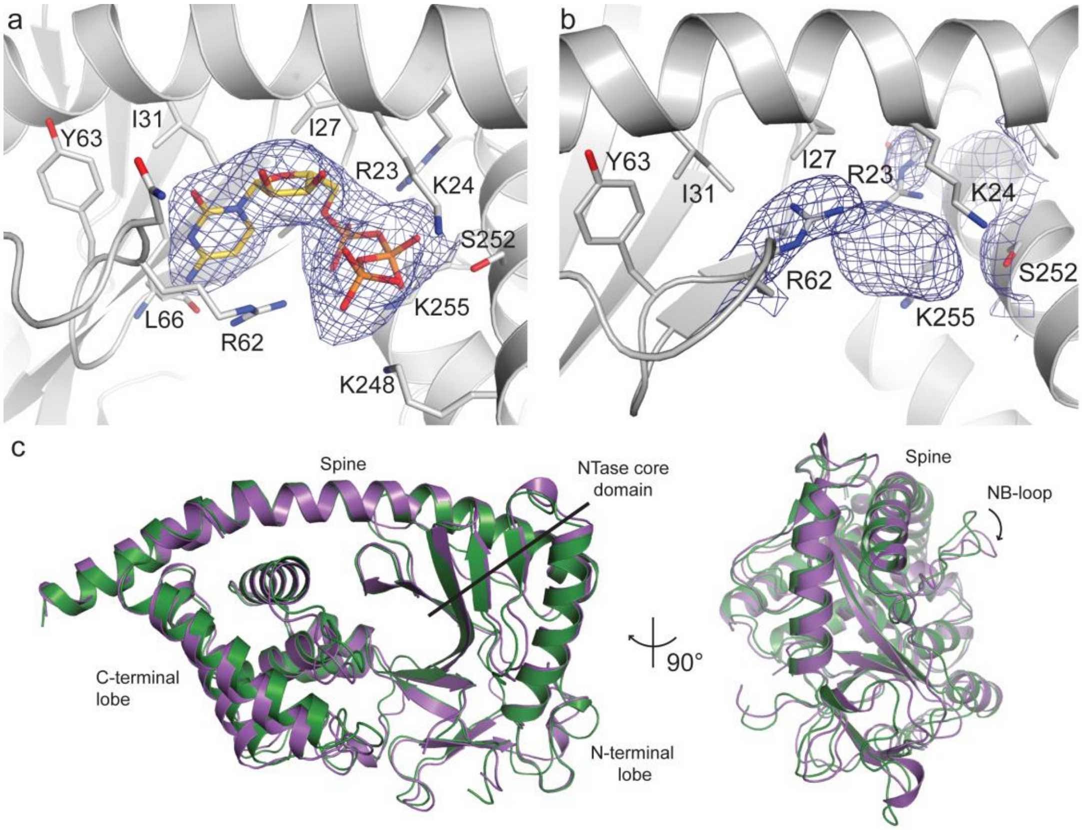

Supplementary figure S3: Composite omit electron density maps for MAB21L1:CTP and unknown ligand in apoMAB21L1 structure, superposition of apoMAB21L1 and MAB21L1:CTP

(a) Composite omit electron density maps of the ligand binding pocket of MAB21L1:CTP and (b) *apo* MAB21L1 contoured at 1σ. The *apo* MAB21L1 structure shows additional density for an unknown ligand. Key residues interacting with the CTP are depicted and annotated. (c) Front and side views of MAB21L1:CTP (purple) superposed with *apo* MAB21L1 (green) with an RMSD of 0.94Å.

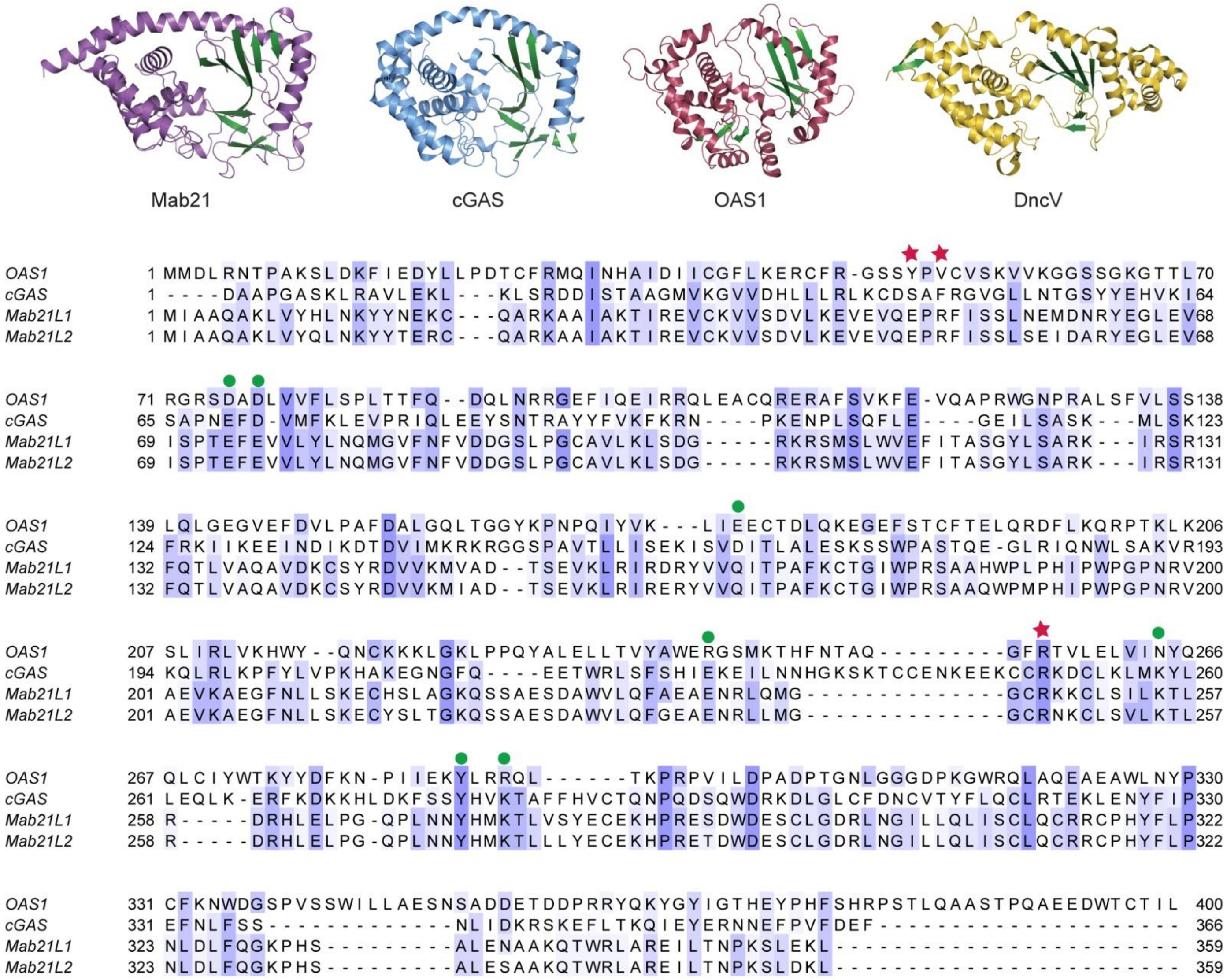

Supplementary figure S4: Structures of the MAB21L1-related proteins cGAS, OAS1 and DncV and a sequence alignment of OAS1, cGAS, MAB21L1 and MAB21L2

Upper panel: crystal structures of MAB21L1 (purple, PDB code 5EOM) and the structurally-related proteins cGAS^{MAB21} (blue, PDB code 4JLX), OAS1 (red, PDB code 4RWQ) and DncV (yellow, PDB code 4XJ1). Lower panel: amino acid sequence alignment of human OAS1, cGAS^{MAB21}, MAB21L1 and MAB21L2 generated with MUSCLE [1] and colored according to the BLOSUM62 conservation score (30%). Green dots mark conserved active site residues involved in metal coordination and conserved residues known to be involved in donor NTP coordination. Red stars denote residues that are mutated in patients with eye malformations in MAB21L2.

1 Edgar, R. C. MUSCLE: multiple sequence alignment with high accuracy and high throughput. *Nucleic acids research* **32**, 1792-1797 (2004).
